# When ‘Dirty’ Drugs Become Useful: Peptide-Guided Exposure Engineering for the Repurposing of Cancer Drugs

**DOI:** 10.3390/ijms27052400

**Published:** 2026-03-05

**Authors:** Serena Marchiò

**Affiliations:** 1 Department of Oncology, University of Turin, 10060 Candiolo, Italy; serena.marchio@ircc.it; Tel.: +39-01199333239; 2 Candiolo Cancer Institute, Fondazione del Piemonte per l’Oncologia—Istituto di Ricovero e Cura a Carattere Scientifico (FPO-IRCCS), 10060 Candiolo, Italy

**Keywords:** cancer, drug repurposing, drug delivery, peptide–drug conjugates, peptide-functionalized nanoparticles, tumor targeting

## Abstract

Drug repurposing in oncology is often framed as a drug–target matching exercise, yet many candidates with plausible biological rationales fail in the clinic. In solid tumors, therapeutic outcomes are constrained not only by pharmacological target relevance but also by limited tumor accessibility, heterogeneous intratumoral exposure, loss of context-dependent activity, and dose-limiting systemic toxicity. This perspective argues that repurposing strategies should treat exposure engineering as a design principle alongside molecular selectivity. Peptides that bind cell- or matrix-associated molecules at the tumor site have the potential to implement spatial, temporal, and subcellular control over where and when a drug engages its pharmacological target, thereby enabling confinement of polypharmacology to tumor contexts. Mechanistic modes of peptide-enabled exposure selectivity (homing, anchoring/retention, conditional activation, penetration enhancement, and subcellular biasing), key failure modes, and translational constraints are discussed, together with an exposure-centric screening workflow to prioritize repurposed agents most amenable to peptide-guided rescue. Emphasizing the combination of exposure control and the addressing-element layer clarifies when and how pharmacologically promiscuous drugs may be repurposed safely and effectively.

## 1. Introduction: Exposure Constraints in Oncology Drug Repurposing

Drug repurposing is usually promoted as a rational shortcut in oncology, because it leverages established safety profiles and known mechanisms to accelerate clinical translation [1]. In this perspective, repurposing is used in its broadest sense, encompassing both the redeployment of oncology agents across tumor contexts and the repositioning of non-oncological drugs whose pharmacological activities may be therapeutically relevant in cancer.

Despite compelling biological rationales, many repurposed candidates fail to achieve meaningful efficacy in solid tumors. These failures are often attributed to insufficient pharmacological target relevance or limited mechanistic novelty, yet a substantial fraction of repurposing efforts appear to stall for reasons that are not purely biological, but pharmacological [1,2]. In solid tumors, therapeutic outcomes are shaped by spatial and temporal constraints that are poorly captured by conventional repurposing pipelines. Frequently, limited tumor accessibility—imposed by vascular, stromal, and architectural barriers—precedes and constrains drug exposure. As a result, systemic administration does not guarantee effective exposure within the most relevant tumor compartments, even when pharmacological targets are present. In addition, heterogeneous perfusion, complex tissue architecture, and dynamic microenvironmental states at the tumor site frequently decouple drug mechanism from effective in vivo activity, even when target expression is preserved. Recent mechanistic syntheses further underscore that delivery to solid tumors is governed by multiple, context-dependent transport modes, rather than a single universal enhanced permeability and retention (EPR) mechanism [3,4,5].

**Figure 1:**
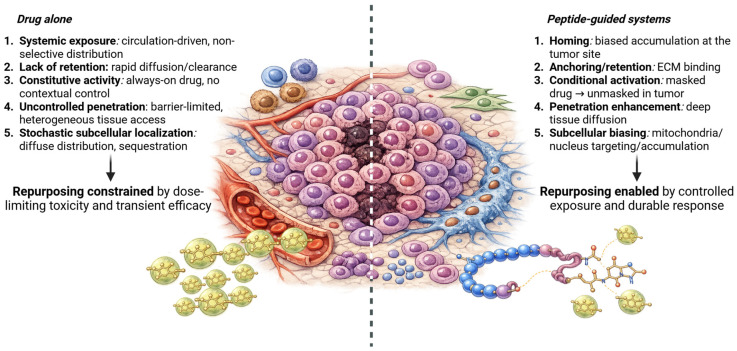
Exposure-guided framework for peptide-enabled drug repurposing in solid tumors. Systemically administered drugs often fail in solid tumors due to non-selective exposure, rapid clearance, loss of contextual activation, and heterogeneous intratumoral access, resulting in dose-limiting toxicity and transient efficacy. Peptide-guided systems introduce an “addressing-element” layer that enables spatial, temporal, and subcellular control over where and when a drug engages its pharmacological target. Mechanistic modes of peptide-guided exposure selectivity include homing, anchoring/retention, conditional activation, penetration enhancement, and subcellular biasing. By reshaping exposure rather than drug chemistry, peptide-guided strategies can enable the safe and effective repurposing of pharmacologically promiscuous drugs in solid tumors.

This perspective argues that drug repurposing strategies should be expanded beyond simple drug–target matching to explicitly address drug exposure as a determinant of therapeutic success (Figure 1). Controlling where, when, and for how long a drug is active can be as decisive as controlling what it binds [3,6].

Within this framework, peptide–drug conjugates and peptide-guided nanocarrier systems provide practical means to impose spatial and conditional selectivity on pharmacologically active compounds. By reshaping exposure rather than drug chemistry, peptide-guided systems offer an opportunity to reconsider repurposed agents that fail under conventional systemic administration [1,7].

In this perspective, the term ‘pharmacological target’ refers to the molecular entity responsive to the drug (e.g., DNA, an enzyme, or a signaling pathway), whereas ‘addressing element’ denotes a ligand peptide that recognizes cell- or matrix-associated moieties—including receptors, extracellular matrix (ECM) motifs, or stromal markers—and is exploited to control where, when, and in which cellular contexts a drug engages its pharmacological target.

## 2. “Dirty” Does Not Mean “Useless”: Redefining Polypharmacology in Solid Tumors

Polypharmacology, or pharmacological promiscuity, is traditionally viewed as a liability in oncology drug development. Compounds that engage multiple pharmacological targets are often deprioritized because systemic exposure translates broad target engagement into widespread physiological perturbation and toxicity [8,9].

In the context of solid tumors, the relationship between polypharmacology and therapeutic failure is more nuanced. Tumors are heterogeneous and adaptive systems characterized by pathway redundancy, clonal diversity, and dynamic interactions with the surrounding microenvironment [10,11]. Under these conditions, narrowly selective agents often produce transient responses, as compensatory signaling and phenotypic plasticity enable rapid escape [10,12]. Conversely, simultaneous perturbation of multiple tumor-relevant pathways (an intrinsic property of many repurposed drugs) can exert more robust pressure on tumor networks, provided that such activity is spatially constrained [8].

Thalidomide and its immunomodulatory derivatives, for example, exert pleiotropic effects on immune regulation, angiogenesis, and cereblon-dependent protein degradation, yet have demonstrated durable clinical benefit in hematologic malignancies [13]. Likewise, multi-kinase inhibitors such as sorafenib achieve anti-tumor activity through simultaneous perturbation of RAF, VEGFR, and PDGFR signaling, reflecting network-level rather than single-target suppression. In repurposing contexts, agents such as metformin, itraconazole, or disulfiram similarly display multi-pathway or stress-network modulation that is biologically plausible in tumors but frequently constrained by exposure limitations in solid tissues [14,15,16].

This distinction motivates a shift from viewing polypharmacology as an intrinsic defect to considering it a context-dependent property. When broad pharmacological target engagement occurs systemically, it manifests as toxicity; when confined to tumor tissues or specific microenvironmental states, it may amplify therapeutic impact while limiting off-target effects [8,9]. Importantly, this reframing does not negate the value of molecular selectivity, but highlights its limitations as a sole determinant of therapeutic index. Many repurposed drugs occupy this intermediate space: mechanistically active but clinically constrained by systemic exposure. Rather than excluding such compounds a priori, exposure-engineering strategies may create an opportunity for their clinical application [8,9].

Several clinically used or extensively investigated drugs illustrate this tension between network-level activity and exposure limitation. Agents such as metformin, itraconazole, disulfiram, and niclosamide exert multi-pathway or stress-network effects that are mechanistically relevant to tumor biology, yet their repositioning in solid tumors has been constrained by pharmacokinetic variability, systemic toxicity, limited bioavailability, or context-dependent activation. Representative examples and their dominant exposure liabilities are summarized in Table 1.

Under conditions of spatial or contextual restriction, pharmacological promiscuity may amplify therapeutic pressure within tumor ecosystems while limiting systemic toxicity. In this sense, “dirty” does not imply biologically irrelevant, but rather exposure-dependent.

## 3. From Molecular Selectivity to Exposure Selectivity

In solid tumors, therapeutic efficacy is governed not only by pharmacological target engagement but also by the spatial and temporal distribution of drug activity. Conventional repurposing approaches implicitly assume that systemic administration provides sufficient access to tumor tissues and relevant cellular states. In practice, however, drug concentration, residence time, and activation vary markedly across tumor compartments, frequently limiting efficacy even when pharmacological targets are present [3,6,18].

Exposure engineering refers to the deliberate control of where, when, and for how long a drug exerts biological activity. Rather than focusing exclusively on delivery efficiency, this concept emphasizes exposure selectivity, namely, the preferential confinement of pharmacological action to tumor tissues or tumor-associated contexts. From this perspective, therapeutic index emerges as a composite property shaped by both molecular selectivity and exposure selectivity, rather than by target specificity alone [19].

A critical distinction must be drawn between exposure engineering and delivery enhancement. Increased uptake or tumor accumulation does not necessarily translate into meaningful selectivity if active drug remains broadly distributed or constitutively active. By contrast, exposure engineering imposes constraints on drug action, biasing pharmacological effects toward defined tissues, cellular populations, or environmental conditions. This distinction is especially relevant for repurposed drugs, whose systemic toxicities often reflect widespread pharmacological target engagement rather than incompatibility with tumor biology [20] (Figure 1).

The distinction between tumor accumulation and exposure selectivity is not merely conceptual. Quantitative analyses of nanoparticle delivery have demonstrated that increased tumor-associated accumulation does not necessarily translate into proportional intracellular drug exposure or therapeutic benefit, particularly in the presence of stromal sequestration and heterogeneous perfusion [15]. Conversely, strategies that alter compartment access—such as peptide-mediated transport across the blood–brain barrier in central nervous system malignancies—illustrate how modifying exposure topology, rather than simply increasing total tumor uptake, can enable pharmacological activity in otherwise inaccessible tumor niches. These observations reinforce that delivery efficiency and spatial pharmacodynamics are not interchangeable metrics.

Peptide-guided exposure engineering strategies are implemented through peptide–drug conjugates, peptide-functionalized nanoparticles, or peptide-assisted co-administration approaches; however, the present perspective focuses on their shared functional logic rather than on specific delivery formats.

Peptides represent a uniquely versatile class of exposure-modulating elements. Their small size, modularity, and chemical flexibility enable precise control over biodistribution, retention, and activation without altering the core pharmacophore. Peptides can be deployed either as direct drug conjugates or as functional components of nanocarrier systems, extending exposure engineering strategies across diverse classes of repurposed agents [7,21]. Recent design-focused analyses emphasize that successful clinical translation depends on linker chemistry, stability–clearance tradeoffs, and quantitative validation of on-target activation, rather than on targeting claims alone [22].

Alternative exposure-modifying platforms, including antibody–drug conjugates, polymer conjugates, liposomal formulations, and small-molecule prodrugs, have also demonstrated the capacity to reshape biodistribution and activation profiles. Antibody-based systems offer high molecular specificity but are constrained by size, tumor penetration limits, and prolonged systemic half-life. Nanoparticle and liposomal carriers can enhance accumulation but often rely on variable transport mechanisms in solid tumors. Small-molecule prodrugs provide chemical conditionality but are less modular once synthesized. In contrast, peptides combine small size, synthetic modularity, tunable pharmacokinetics, and compatibility with diverse payload classes, allowing implementation of homing, retention, conditional activation, penetration, and subcellular biasing within a unified design logic. This functional flexibility, together with synthetic accessibility and tunable pharmacokinetics, motivates their focused consideration in the present Perspective without excluding the relevance of alternative exposure-modulating platforms.

Importantly, exposure engineering does not seek to compensate for weak or irrelevant drug mechanisms. Instead, it provides a rational means to reconcile mechanistically active but clinically constrained compounds with the spatial and contextual realities of solid tumors. Elevating exposure selectivity to a first-class design consideration opens opportunities to reinterpret pharmacological breadth as a conditional advantage [3].

## 4. Mechanistic Modes of Peptide-Guided Exposure Selectivity

Peptide-guided systems achieve exposure selectivity through a limited set of recurring mechanistic strategies. While individual implementations differ, most peptide–drug conjugates and peptide-functionalized nanoparticles bias drug distribution, retention, activation, or intracellular routing in predictable ways. Recognizing these modes provides a conceptual framework for matching repurposed drugs to appropriate peptide designs [7,23] (Figure 1). Although these mechanistic modes are presented separately, they are defined by the primary exposure variable they modify: homing alters macroscopic tissue distribution; anchoring/retention extends local residence time; conditional activation restricts pharmacological activity; tumor penetration modifies intratumoral spatial distribution; and subcellular biasing redirects intracellular localization. While individual systems may combine multiple mechanisms, distinguishing their dominant functional effect clarifies design logic and prevents conceptual overlap.

### 4.1. Homing: Biasing Drug Accumulation

Homing peptides promote preferential accumulation of drug payloads in tumor tissues by recognizing receptors, surface proteins, or extracellular matrix components enriched in the tumor milieu [24]. Here, the addressing element is the peptide; receptors or matrix features are the recognized moieties that confer addressability. Homing strategies are most effective when the recognized moiety is sufficiently abundant, accessible, and spatially restricted, enabling meaningful enrichment relative to normal tissues [7,25,26].

Clinically relevant precedents illustrate how peptide-mediated homing can overcome otherwise prohibitive delivery barriers [27]. For example, peptide–drug conjugates incorporating angiopep-2 have demonstrated the ability to transport cytotoxic payloads across the blood–brain barrier, enabling therapeutic exposure in central nervous system tumors and metastases that are largely inaccessible to systemically administered small molecules [28]. This case highlights how peptide-guided homing can unlock tumor compartments rather than simply increase overall exposure.

For repurposed drugs, homing peptides can reduce the systemic dose required to achieve local pharmacological activity. However, this strategy remains sensitive to heterogeneity and accessibility of the recognized moiety, receptor shedding, and limited penetration beyond perivascular regions. As a result, homing alone rarely provides sufficient exposure selectivity and often benefits from combination with mechanisms that enhance retention or restrict activation.

### 4.2. Anchoring and Retention: Extending Local Residence Time

Anchoring peptides enhance exposure selectivity by promoting retention within tumor tissues, often through interactions with extracellular matrix or stromal components. Rather than maximizing peak uptake, this strategy prolongs local residence time, effectively creating intratumoral drug depots [29].

This mode is particularly attractive for repurposed drugs limited by rapid clearance or transient pharmacological target engagement. However, excessive stromal sequestration may impede access to tumor cells, underscoring the need to balance retention with controlled release and diffusion [30].

### 4.3. Conditional Activation: Context-Dependent Drug Unmasking

Conditional activation represents one of the most powerful mechanisms for achieving exposure selectivity. In this mode, peptides function as molecular gates, masking drug activity until specific tumor-associated conditions—such as elevated protease activity, acidic pH, redox imbalance, or hypoxia—are encountered. By decoupling biodistribution from pharmacological activation, conditional strategies can limit off-target effects even when systemic exposure persists [31,32].

Conditional activation is particularly relevant for repurposed drugs whose clinical limitations arise from systemic pharmacological target engagement rather than lack of intrinsic anti-tumor activity. Protease-cleavable peptide linkers and masking motifs, including those used in conditionally activated prodrugs and masked biologics, exemplify how enzymatic activity within the tumor microenvironment can be exploited to spatially restrict drug action [31,33]. These strategies illustrate that exposure selectivity can be achieved even in the absence of strict tumor-specific accumulation.

At the same time, conditional activation is highly sensitive to trigger choice and validation. Tumor-associated cues must be sufficiently robust, spatially restricted, and reproducible to confer meaningful selectivity. Misalignment between trigger distribution and therapeutically relevant tumor cell populations can result in incomplete activation or unintended off-tumor effects, emphasizing the need for quantitative validation of conditionality rather than reliance on nominal tumor specificity [31].

### 4.4. Penetration Enhancement: Expanding Intratumoral Reach

Penetration-enhancing peptides facilitate transport across vascular, stromal, and cellular barriers, improving access to poorly perfused or densely packed tumor regions. In this mode, exposure selectivity arises from improved intratumoral distribution rather than preferential tumor accumulation. Such strategies are particularly relevant for repurposed drugs whose failure reflects limited access to tumor cells despite adequate systemic exposure [6].

Tumor-penetrating peptides such as iRGD provide a well-characterized example of this principle [34,35]. Next-generation tumor-penetrating strategies increasingly incorporate protease-activated mechanisms to limit off-tumor accumulation while preserving deep-tissue transport [36]. By engaging tumor-associated receptors and activating trans-tissue transport pathways, these peptides have been shown to enhance intratumoral penetration of co-administered drugs and nanocarriers in preclinical models [37]. Importantly, this effect can be achieved without permanent conjugation, underscoring penetration as a separable design dimension from homing or activation.

However, penetration enhancement carries inherent risks. Improved access to tumor tissues may be accompanied by increased uptake in non-tumor compartments unless combined with additional selectivity mechanisms. Consequently, penetration-focused strategies are most effective when integrated with homing or conditional activation to preserve spatial control over pharmacological activity [6,35].

### 4.5. Subcellular Biasing: Steering Pharmacological Output

Subcellular targeting peptides introduce an additional layer of exposure selectivity by directing payloads to specific intracellular compartments. By altering intracellular compartmentalization, subcellular biasing can modify the balance of downstream signaling outputs and stress responses associated with multi-target drugs. For repurposed drugs with pleiotropic mechanisms, subcellular biasing offers a means to prioritize tumor-relevant effects. However, success depends on precise control of trafficking and release kinetics [38,39]. Clinical experience with peptide–drug conjugates and related exposure-engineered systems demonstrates that mechanistic plausibility alone is insufficient. Durable benefit depends on quantitatively validated spatial pharmacodynamics and therapeutic index improvement, reinforcing the need for disciplined mechanism matching. The relevance of each mechanistic mode depends on the specific exposure liability of the repurposed drug.

## 5. Matching Repurposed Drug Archetypes to Peptide-Guided Strategies

Peptide-guided exposure engineering is most effective when applied selectively to repurposed drugs whose failure reflects exposure-related constraints rather than inadequate biological relevance. Distinguishing between these scenarios is therefore a critical first step in rational prioritization. For the purposes of this framework, drugs are assigned to archetypes based on the dominant exposure-related constraint documented in preclinical or clinical evaluation. Classification is therefore operational rather than mechanistic: the same compound could fall into different categories depending on whether its principal limitation arises from systemic toxicity, context-dependent activation, pharmacokinetic instability, or restricted tumor access.

One common archetype comprises drugs that demonstrate tumor-relevant pharmacodynamic activity at exposures exceeding tolerated systemic levels, resulting in a narrow therapeutic window despite mechanistic plausibility. Typical examples include agents that modulate stress-response or metabolic pathways and show efficacy at concentrations that cannot be safely achieved systemically. For this class, anchoring/retention or conditional activation strategies can preserve pharmacological activity while reducing off-target exposure [19].

A second archetype includes drugs whose anti-tumor effects depend on specific microenvironmental conditions (e.g., hypoxia, oxidative stress, inflammatory signaling) as demonstrated in controlled in vitro or ex vivo systems but attenuated in heterogeneous in vivo contexts. These compounds often perform well in vitro under defined conditions but lose efficacy in vivo when environmental cues are diluted or spatially heterogeneous. Conditional activation strategies are particularly well suited here, as they reintroduce environmental gating and restore alignment between drug mechanism and tumor physiology [10,40].

Polypharmacologic network modulators represent a third archetype. These compounds engage multiple signaling pathways, often confirmed by kinase profiling or systems-level analyses, and are frequently deprioritized due to limited specificity or ambiguous mechanisms of action. However, in tumors characterized by pathway redundancy, such broad engagement can suppress compensatory signaling and phenotypic escape if confined to malignant tissues. Homing and retention strategies can bias their action toward the tumor, converting polypharmacology into localized multi-pathway pressure reducing or avoiding systemic toxicity [8,41].

Finally, pharmacologically potent but poorly behaved compounds, including drugs limited by poor solubility, instability, or unfavorable pharmacokinetics, form a fourth archetype. Such agents demonstrate pharmacological target engagement in vitro but fail to achieve sustained or reproducible tumor exposure in vivo due to rapid clearance, instability, or formulation constraints. Peptide-functionalized nanoparticles can improve physicochemical performance while simultaneously enabling exposure selectivity through homing or conditional activation [7,20].

Across these archetypes, a unifying principle emerges: peptide-guided strategies are most effective when explicitly matched to the reason a repurposed drug failed, rather than applied generically. Exposure engineering should therefore be viewed as a diagnostic-driven design process, where pharmacological limitations inform peptide selection and mechanism, providing a rational basis for candidate prioritization.

## 6. Failure Modes and Red Flags in Peptide-Guided Repurposing

Peptide-guided exposure engineering is not universally effective and may introduce new translational challenges. A common pitfall is equating increased tumor uptake with meaningful exposure selectivity; without restricting drug activity, systemic toxicity may persist [20]. Heterogeneity and limited accessibility of the recognized moiety (e.g., receptor or matrix feature) remain major constraints, as do stromal sequestration and perivascular trapping [6,10]. Conditional activation strategies are vulnerable to trigger misalignment, variability, and insufficient specificity [31].

Additional challenges include immunogenicity, altered clearance, toxicity redistribution, and manufacturing reproducibility [7,42]. Peptide functionalization can introduce new epitopes or structural motifs that alter immune recognition, particularly under repeated dosing regimens, thereby limiting durability. Conjugation may also modify volume of distribution, plasma protein binding, or renal filtration profiles, leading to unanticipated pharmacokinetic shifts relative to the parent compound. In some cases, spatial restriction of exposure redistributes rather than eliminates toxicity, shifting adverse effects to previously unaffected compartments. These factors underscore that exposure engineering introduces new pharmacological variables that must be quantitatively characterized rather than assumed to be beneficial.

Clinical experience also illustrates that peptide-based exposure engineering does not guarantee durable benefit. For example, peptide-mediated prodrug strategies that initially showed promise in accelerating tumor-associated activation have, in some cases, failed to demonstrate clinical advantage in confirmatory trials, ultimately leading to regulatory withdrawal [43,44]. Such outcomes underscore the importance of validating not only delivery or activation mechanisms, but also their impact on spatial pharmacodynamics and therapeutic index in clinically relevant settings [3,19].

Collectively, these failure modes reinforce a central principle: peptide-guided repurposing is justified only when it produces demonstrable and durable changes in spatiotemporal pharmacology. Without clear evidence of exposure restriction or context-dependent activation, peptide modification risks adding complexity without therapeutic gain.

Several recurring pitfalls should be explicitly avoided when applying peptide-guided exposure engineering to drug repurposing:

1. Do not equate increased tumor uptake with exposure selectivity. Without evidence of restricted pharmacological activity, higher accumulation alone does not imply improved therapeutic index.
2. Do not add addressing elements without diagnosing the dominant failure mode. Peptide functionalization should address a specific exposure constraint (e.g., clearance, context loss, penetration), not serve as a generic enhancement.
3. Do not assume that conditional activation is intrinsically tumor-specific. Triggers such as protease activity or acidity must be spatially restricted and quantitatively validated to confer meaningful selectivity.
4. Do not ignore stromal sequestration and compartmental trapping. Retention within extracellular or perivascular compartments may limit tumor cell exposure and reduce efficacy.
5. Do not interpret formulation complexity as therapeutic advancement. Added peptide or nanocarrier features should be justified by demonstrable changes in spatiotemporal pharmacology, not by design sophistication alone.

Successful implementation of peptide-guided exposure engineering typically requires three conditions: (i) the presence of a spatially restricted and sufficiently abundant recognized moiety; (ii) a clear mismatch between systemic exposure and tumor-relevant pharmacodynamics that can be corrected by spatial or conditional restriction; and (iii) quantitative evidence that peptide modification preserves or enhances the intended pharmacological mechanism without introducing dominant new liabilities. In the absence of these conditions, peptide conjugation may increase formulation complexity without improving therapeutic index.

Each of these pitfalls can be mitigated through deliberate translational safeguards. Increased tumor uptake should be coupled with spatially resolved pharmacodynamic measurements to confirm localized target engagement. Addressing elements should be selected only after quantitative diagnosis of the dominant exposure constraint, supported by biodistribution and pharmacokinetic analyses. Conditional activation mechanisms require rigorous validation of trigger abundance, spatial restriction, and reproducibility across patient-derived models. Stromal retention should be balanced against controlled release kinetics to preserve tumor cell access. Finally, manufacturability, immunogenicity risk, and pharmacokinetic shifts introduced by peptide functionalization should be evaluated early to ensure that exposure engineering produces measurable therapeutic index improvement rather than formulation complexity alone.

## 7. A Screening Workflow for Identifying Peptide-Rescuable Repurposed Drugs

Effective implementation of exposure engineering requires its integration into early repurposing workflows rather than its application as a late-stage formulation strategy. Candidate selection should prioritize drugs that exhibit tumor-relevant polypharmacology but are clinically constrained by exposure-related factors, such as dose-limiting toxicity, rapid clearance, or loss of context-dependent activity. Repurposed agents lacking a credible biological rationale are unlikely to benefit from peptide-guided strategies and should be excluded [1,45]. At this stage, curated resources that catalog peptide–drug conjugate architectures, linkers, payloads, and biological activity can support evidence-based prioritization and reduce empirical trial-and-error [46].

Once candidates are identified, the dominant exposure-related failure mode should be explicitly diagnosed. Drugs may fail due to insufficient tumor accumulation, limited intratumoral retention, dilution of microenvironmental cues, or restricted access to relevant tumor compartments. Defining this bottleneck is essential, as different peptide mechanisms—homing, anchoring, conditional activation, penetration enhancement, or subcellular biasing—address distinct constraints and are not functionally interchangeable. Applying addressing elements without such diagnosis risks adding complexity without altering the underlying pharmacological limitation.

Evaluation of candidate repurposed drug–peptide combinations should rely on context-preserving experimental systems that capture spatial heterogeneity and microenvironmental gradients, such as three-dimensional co-cultures, tumor organoids incorporating stromal components, or perfused models [47]. Importantly, biodistribution measurements should be coupled with spatially resolved pharmacodynamic readouts to determine whether altered exposure translates into localized biological activity, rather than relying on delivery metrics alone [3,18,48]. Emerging spatial-omics frameworks further provide practical guidance for integrating spatially resolved measurements into clinically oriented study design and response assessment [49].

Advancement decisions should be guided by explicit, exposure-centric criteria that are prospectively defined rather than retrospectively interpreted. Evidence of successful peptide rescue should include demonstrable spatial restriction of drug activity, such as reproducible improvement in tumor-to-normal pharmacodynamic signal, sustained local target engagement relative to systemic dosing, or quantifiable trigger-dependent activation when conditional systems are used. Importantly, increased tumor accumulation alone is insufficient; validation should demonstrate that altered biodistribution translates into measurable improvement in therapeutic index proxies compared with the unconjugated drug. Wherever feasible, exposure selectivity should be evaluated using spatially resolved pharmacodynamic assays, compartment-specific drug quantification, or paired pharmacokinetics–pharmacodynamic modeling to ensure that advancement decisions are anchored in quantitative evidence rather than delivery metrics alone [19,20].

**Figure 2:**
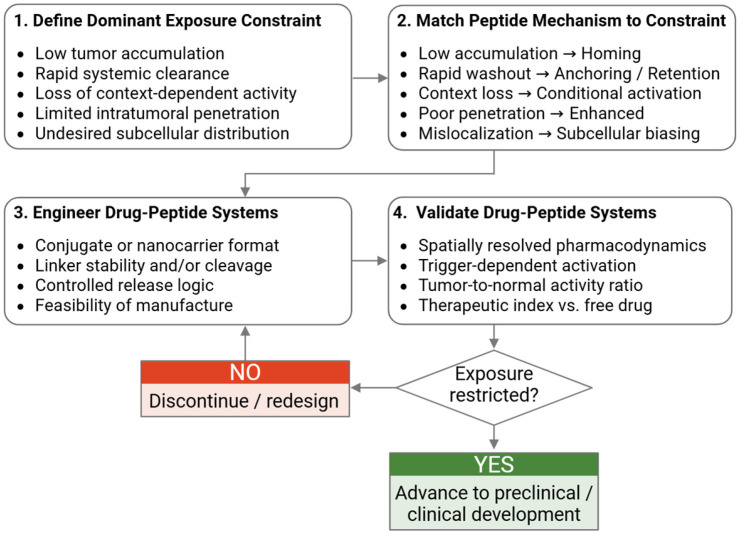
Exposure-centric workflow for identifying peptide-rescuable repurposed drugs. Drug candidates with tumor-relevant polypharmacology are first evaluated to define the dominant exposure constraint limiting therapeutic efficacy (e.g., insufficient accumulation, rapid clearance, context loss, limited tumor penetration, or undesired subcellular distribution). Peptide mechanisms are then selected to directly address the identified liability (homing, anchoring/retention, conditional activation, penetration enhancement, or subcellular biasing). Engineered drug–peptide systems are constructed with attention to conjugation format, linker stability or cleavage, controlled release, and manufacturability. Advancement requires quantitative validation of spatial pharmacodynamics, trigger-dependent activation when applicable, improvement in tumor-to-normal activity ratio, and enhanced therapeutic index relative to the free drug. Only candidates demonstrating restricted exposure with preserved pharmacological activity proceed to further development.

Taken together, this workflow emphasizes that peptide-guided repurposing is an ensemble design problem, in which drug, peptide, and nanocarrier (when present) function as an integrated system. Success depends not on maximizing design sophistication, but on aligning pharmacological mechanism with spatiotemporal exposure constraints. Embedding these principles into repurposing workflows provides a disciplined yet flexible framework to reassess compounds previously deemed clinically impractical, while maintaining rigor in translational decision-making. This exposure-centric decision framework is summarized schematically in Figure 2. In the absence of reproducible exposure restriction with preserved mechanism, peptide modification should not advance, regardless of formulation sophistication.

## 8. Conclusions and Outlook: Repurposing by Exposure Restriction

Drug repurposing in oncology has largely emphasized target reassignment while underestimating the constraints imposed by tumor exposure. This Perspective argues that many repurposed drugs fail not because of inadequate biological mechanisms, but because systemic administration fails to align pharmacological activity with the constraints of tumor tissues. Incorporating exposure selectivity alongside molecular selectivity provides a complementary framework for understanding these failures and for identifying opportunities for rescue.

Peptide-guided systems offer a versatile means to engineer drug exposure without altering core pharmacophores, enabling spatial restriction, conditional activation, and controlled intracellular routing. Within this framework, pharmacological promiscuity (commonly viewed as an intrinsic liability) can, under defined conditions, be transformed into localized multi-pathway pressure that is better suited to heterogeneous and adaptive tumor ecosystems. Importantly, this approach does not replace drug design, but rather delineates the conditions under which exposure control and the addressing-element layer can reconcile mechanistic activity with acceptable therapeutic index.

Looking forward, broader adoption of exposure-aware repurposing will require changes in both experimental practice and evaluative criteria. Greater emphasis on spatial pharmacodynamics, context-preserving models, and explicit exposure-centric go/no-go decisions will be essential to distinguish meaningful rescue from superficial delivery enhancement. Framing repurposing as an ensemble design problem—integrating drug, peptide, and carrier when appropriate—offers a disciplined yet flexible strategy to reassess compounds previously deemed clinically impractical. In doing so, exposure engineering may expand the actionable space of drug repurposing while maintaining rigor in translational decision-making.

## Figures and Tables

**Table 1:** Representative clinically used “dirty” drugs with tumor-relevant polypharmacology and dominant exposure liabilities in solid tumors.

Drug (Original Use)	Tumor-Relevant Actions	Key Limitation	Ref.
Metformin (type 2 diabetes)	Metabolic stress; mTORC1 suppression	Context-dependent efficacy; systemic exposure constraints	[14]
Itraconazole (antifungal)	Hedgehog inhibition; anti-angiogenic effects	PK variability; dose-limiting toxicity at high exposure	[15]
Disulfiram (alcohol aversion)	Cu-dependent proteostasis disruption	Active metabolite formation	[16]
Niclosamide (anthelmintic)	Wnt/β-catenin inhibition	Poor solubility; limited bioavailability	[17]
Thalidomide (immunomodulatory)	Immunomodulatory; anti-angiogenic	Systemic toxicity; narrow therapeutic window	[13]

## Data Availability

No new data were created or analyzed in this study. Data sharing is not applicable to this article.

## Abbreviations

The following abbreviations are used in this manuscript:

ECM	Extracellular matrix
EPR	Enhanced permeability and retention
